# Seroprevalence and Risk Factors Associated with Canine Leishmaniasis in Egypt

**DOI:** 10.3390/vetsci8100236

**Published:** 2021-10-15

**Authors:** Abdelfattah Selim, Salma Shoulah, Abdelhamed Abdelhady, Abdulaziz Alouffi, Yasser Alraey, Waleed S. Al-Salem

**Affiliations:** 1 Department of Animal Medicine (Infectious Diseases), College of Veterinary Medicine, Benha University, Toukh 13736, Egypt; salma.soulah@fvtm.bu.edu.eg; 2 Department of Parasitology and Animal Diseases, National Research Center, Giza 8655, Egypt; afanrc@yahoo.com; 3 King Abdulaziz City for Science and Technology, Riyadh 12354, Saudi Arabia; asn1950r@gmail.com; 4 Chair Vaccines Research of Infectious Diseases, King Saud University, Riyadh 11495, Saudi Arabia; 5 Department of Clinical Laboratory Sciences, College of Applied Medical Sciences, King Khalid University, Abha 62529, Saudi Arabia; yahamd@kku.edu.sa; 6 Department of Parasitology, Liverpool School of Tropical Medicine, Liverpool L3 5QA, UK; Waleed-alsalem@hotmail.com; 7 Minister of Environment, Water and Agriculture, Riyadh 11195, Saudi Arabia

**Keywords:** *L. infantum*, dogs, ELISA, Egypt

## Abstract

Background: Canine leishmaniasis (CanL) is caused by *Leishmania infantum* (*L. infantum*) that is transmitted by sand fly vectors with dogs acting as the main reservoir. Methods: The present study aimed to determine the seroprevalence of CanL in dogs from Egypt and assessed the associated risk factors. The study was conducted from 2019 to 2020 in five governorates situated in Northern Egypt. Serum samples from 450 asymptomatic dogs were serologically examined by use of enzyme-linked immunosorbent assay (ELISA). Results: Overall, the seroprevalence rate of CanL was 21.3% and the highest rates were observed in Cairo and Giza governorates. The univariable analysis revealed that the seropositivity of CanL was strongly related to the dogs’ ages, length of hair, absence of veterinary care or application of insecticides, and the type of floor of their shelters. The risk factors that were found to be associated with CanL in exposed dogs were: age group 2–4 years old (OR = 12, 95% CI: 1.6–92.3); short hair (OR = 2.07, 95% CI: 1.2–3.6); absence of veterinary care (OR = 2.7, 95% CI: 1.3–5.8); no application of insecticides (OR = 3.09, 95% CI: 1.5–6.5) and their residence in a shelter with an earthen floor (OR = 1.42, 95% CI: 0.7–2.9). Conclusions: Based on the present results, CanL is present in Egyptian dogs and this increases the possibility of transmission by sand fly to humans with whom they have contact. Consequently, an efficient monitoring programme and effective control measures are important to reduce the risk of infection.

## 1. Introduction

Canine leishmaniasis (CanL) is a neglected zoonotic disease that is caused by *Leishmania* spp. [1]. Leishmaniasis is a vector-borne disease that is caused by protozoan flagellates. It affects several animal species including humans and is transmitted via bites of the infected female *Phlebotomus* sand fly [2,3].

The disease is endemic in tropical and temperate regions in 98 countries, and visceral leishmaniasis (VL) is the most fatal and common form in the Mediterranean region [4,5]. Zoonotic leishmaniasis caused by *Leishmania infantum* (*L. infantum*) is endemic in most of the Middle East, North Africa and the Mediterranean, including Egypt [6].

Domestic dogs are the main reservoir for leishmaniasis and can become infected at any age [7]. The clinical features of CanL vary from asymptomatic through self-limiting to severe viscero-cutaneous infection [8,9]. However, asymptomatic dogs can develop clinical signs during their lives, while *L. infantum* can spread unnoticed within the dog population and remain as a source of infection for sand flies, which transmit the parasite to other hosts [10]. Hence, early diagnosis of these asymptomatic carriers is critical for disease control in both endemic and non-endemic countries [11].

Leishmaniasis was reported in Egypt over 4000 years ago, in ancient Egyptian mummies. In past decades, *L. major* has been detected in dogs from Egypt [12] and antibodies against *Leishmania* spp. were detected in dogs from Egypt by Morsy, et al. [13]. In addition, there have been several reports from the mid to late 20th century of sporadic cases of cutaneous leishmaniasis (CL) and VL among Egyptian people, particularly those in the Suez Canal, Sinai and Agamy regions in Alexandria [4,14,15,16,17,18]. Recently, the presence of *L. tropica* in humans and *L. infantum* in dogs has been proven [1].

Serological examination is a useful tool to detect specific antibodies and to determine the spread of the disease, because a large proportion of dogs are asymptomatic [19]. Enzyme-linked immunosorbent assay (ELISA) and indirect fluorescent antibody tests (IFAT) are the most widely used serological methods [20]. ELISA shows potential as a sensitive tool for mass screening in epidemiological studies and is suited to field conditions [21,22].

In Egypt, the prevalence of *L. infantum* in dogs is uncertain, especially with changes of climatic conditions, dog populations and distributions of sand flies, all of which affect the epidemiology of the disease.

To fill this knowledge gap, the present study aimed to determine the seroprevalence of CanL and the risk factors that were associated with infection among dogs in some governorates in Northern Egypt.

## 2. Materials and Methods

### 2.1. Ethics Statement

All procedures involving the handling and collection of samples from dogs used in this study were approved by the ethical committee for Animal Experiment of Benha University and informed consent was obtained from owners. All methods regarding animals and human participant in the study were performed in accordance with the relevant guidelines and regulations and were approved by ethical committee of faculty of veterinary medicine, Benha University.

### 2.2. Study Area

**Figure 1:**
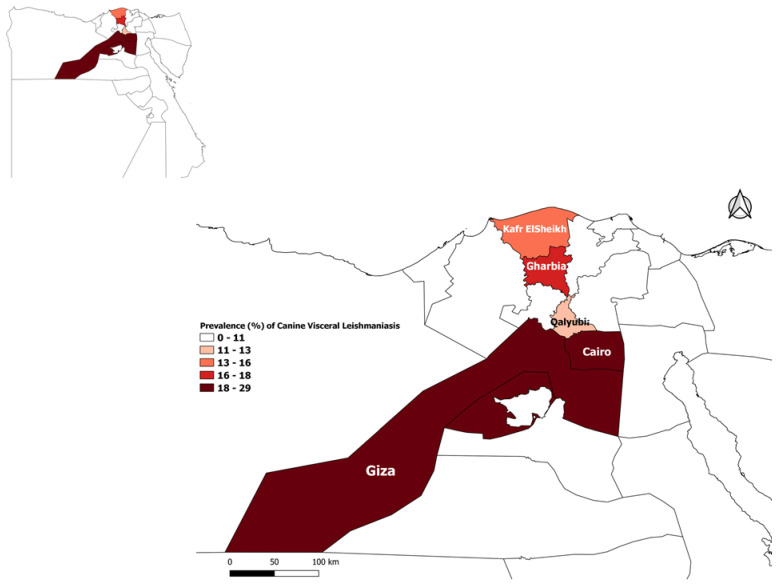
Map of Egypt showing governorates that were considered in the study.

The study was conducted during the period from June 2019 to May 2020 in five governorates located in Northern Egypt. These governorates were Cairo (30.0444° N, 31.2357° E), Giza (30.0131° N, 31.2089° E), Qalyubia (30.3292° N, 31.2168° E), Kafr ElSheikh (31.1107° N, 30.9388° E) and Gharbia (30.8754° N, 31.0335° E), Figure 1. The climate of the selected areas is Mediterranean with dry, hot summers and wet winters.

### 2.3. Sampling and Data Collection

The required samples size was determined using Danial′s formula as follows:

(1) $$n=\frac{z^{2}p(1-P)}{d^{2}}$$

where *n* is number of appropriate sample, *z* is level of confidence (*z* value is 1.96 if 95% confidence level is conventional), *P* is prevalence level which was 10% based on previously study of [12] and e is precision whereas this study’s precision (e) was 5% based on [23]. According to dog population in each examined area and based on Danial’s formula, the estimated sample size from each area was 95, 115, 75, 80 and 85 from Cairo, Giza, Qalyubia, Kafr ElSheikh and Gharbia governorates.

During June 2019 to May 2020, a total of 450 blood samples (2 mL) were collected from the saphenous or cephalic veins of dogs that had been admitted to veterinary clinics distributed across the five governorates. Serum samples were separated by centrifugation at 3500× *g* for 10 min and preserved at −20 ℃ for serological examination. In addition, the data for each dog were gathered when the samples were collected. These data included location of the dog, its age, sex and hair length. Additionally, owners were questioned regarding the veterinary care they supplied to the dog, whether any insecticides had been applied to the animals against sand flies and the type of floor on which they slept.

### 2.4. Statistical Analysis

The study data were analysed through use of the statistical package for the social sciences (SPSS) software (ver. 24.0, IBM, Endicott, NY, USA). A chi-square test was applied to compare seropositivity to each variable for *L. infantum* and the results were considered significant if *p* was ≤0.05. Univariate logistic regression was performed to determine any association between the seropositivity of exposed dogs for *L. infantum* and variables of location (five governorates), age (6–12 months, 1–2 years, 2–4 years, 4–6 years or >6 years old), sex (male or female), hair length (short or long), floor of shelter (paved or soil), level of veterinary care and whether or not insecticide had been applied. The variables with *p* ≤ 0.2 were included in a multivariable logistic model to determine risk factors, odds ratios (ORs) and confidence intervals (CIs) for each significant variable. The Hosmer–Lemeshow goodness-of-fit test was used to determine the fit of the multivariable logistic regression model.

## 3. Results

A total of 450 blood samples were collected from asymptomatic dogs that were distributed across five governorates of Northern Egypt. In this study, antibodies against *L. infantum* were detected in 96 (21.3%) of 450 examined dogs. In general, there were significant differences (*p* ≤ 0.05) between the different localities that were visited in the study. The seroprevalence rate ranged between 17.6% and 28.4%. The governorates of Cairo (28.4%) and Giza (21.7%) showed the highest rates of infection, Table 1.

According to univariate analysis, the seroprevalence rate increased significantly with exposed dogs in the >2–4-year age group that were most likely to be infected. However, no association (*p* > 0.05) was found between sex and CanL infection.

A further analysis revealed that the seropositive rate was higher among German Shepherd (18.2%, 95% CI: 13.2–24.7) and rottweiler (27%, 95% CI: 19.3–36.4) vs another breed and in short-haired dogs (25%, 95% CI: 20.3–30.4) was higher than that in long-haired dogs, particularly among those raised in earthen-floor shelters (36.4%, 95% CI: 28.5–45). Likewise, the seroprevalence of *L. infantum* was strongly associated with lack of veterinary care (33.7%, 95% CI: 27–40.9) and with no insecticide having been applied (35.6%, 95% CI: 28.7–43.1), Table 1.

Application of a multivariate logistical regression model identified age, hair length, veterinary care, application of insecticides and life in a shelter with earthen floor as definitive predictors of *L. infantum* infection in exposed dogs. According to the multivariate model, the risk of infection increased in middle age, peaking in dogs aged 2–4 years old (OR = 12, 95% CI: 1.6–92.3), male dogs (OR = 1.93, 95% CI: 1.1–3.3) German Shepherd breed (OR = 1.59, 95% CI: 0.9–2.9) and in short-haired dogs (OR = 2.07, 95% CI: 1.2–3.6). Moreover, an absence of veterinary care (OR = 2.7, 95% CI: 1.3–5.8) or no application of insecticides (OR = 3.09, 95% CI: 1.5–6.5) were associated with higher risk of seropositivity. Regarding the floor of the shelter, dogs that lived in earthen-floored shelters seemed to be at higher risk of infection (OR = 1.42, 95% CI: 0.7–2.9), Table 2.

## 4. Discussion

CanL caused by *L. infantum* is an emerging parasitic disease in the Mediterranean region, including Egypt. Dogs form the main reservoir and remain asymptomatic for long periods of their lives, so are considered to be a source of infection for other hosts. Until now, data on CanL in Egypt are fragmented and scarce. This first survey presents a picture of the current epidemiology of CanL in Egypt, including the associated risk factors.

In order to facilitate and improve disease control, sensitive diagnostic tests that may be used in the field are becoming increasingly important. IFATs, ELISAs and direct agglutination tests (DATs) are the most widely used serological tests in CanL screening [14,20]. ELISA is a highly applicable technique that is characterised by high sensitivity, but its specificity depends on the antigen. The specificity and sensitivity of the ELISA test that was employed to detect CanL in this investigation were 99.1% and 98.5%, respectively [21].

Overall, the seroprevalence rate of CanL in exposed dogs was 21.3%, which was in accordance with a previous rate (19.5%) that was reported in the North-Eastern and Pyrenean areas of Spain [19]. In the present study, the seroprevalence rate showed non-significant disparity (*p* = 0.382) between governorates that were visited for the study. The highest prevalence rates among the governorates were observed in Cairo and Giza. In previous studies performed in Egypt, the prevalence rate of CanL was estimated to be 66.6% by polymerase chain reaction [1] and 10% according to immunochromatography [12].

In other studies, the seroprevalence rate of CanL in exposed dogs as measured by ELISA was estimated to be 15.4% in Iran [21], 10.5% in the west of Iran [24], 5.5% in Palestine [25] and 26.6% in Pakistan [26]. Through use of IFAT, the seroprevalence rate was estimated to be 15.4% in Sardinia, Italy [5] and ranged between 42.9% and 74.3% in Sudan [27].

These disparities could be explained by differences in sampling techniques, serological tests, ecological factors and encroachment on urban areas [2,6,12,28,29,30].

In the present study, dogs older than two years were more likely to become infected. This finding was in accordance with those of previous studies, which confirmed that the risk of infection increased with the age of the dogs [20,31,32]. A popular explanation is that adult dogs remain outside for long periods and that increases their chance of contact with vectors [33,34,35,36], and that the immune response against latent infection in resistant dogs may develop in older dogs [37].

Concerning the sex of the dogs, the males showed higher prevalence rates but without significant differences. This finding is consistent with those of [5,37]. This result may be attributable to the roaming behaviour of males [20] or a host immune response that results from the properties of the testosterone hormone in males [38,39,40,41].

CanL susceptibility is known to vary depending on a variety of host-related factors, such as dog breed. German Shephered was more likely to get an infection than other examined breeds. This result ties well with previous studies [42] wherein certain breeds such as German Shephered and Rottweiler are more susceptible to being infected. It has been suggested that the breed’s relative immunocompetence derived from a cellular, parasite-specific immune response, which is linked to clinical wellness [43].

Interestingly, the risk of being seropositive to *L. infantum* increased significantly among short-haired dogs, which was also in agreement with other previous studies [44,45]. Indeed, phlebotomine sand flies are known to feed on hairless areas such as the border of the canine muzzle, which is always exposed [44]. Moreover, long hair decreases emissions of CO_2_ and heat radiation from the host’s body, making it less appealing to vectors [46].

Furthermore, the strong association that was found between the seropositivity of the exposed dogs and the absence of veterinary care or application of insecticides was also as previously reported [5]. This result could be due to the ineffectiveness of control measures such as use of repellent collars or insecticides and the absence of a vaccination protocol against CanL, which is related to a high risk of infection [47].

Another important risk factor that was identified in this study was the material used for the floor of the shelter. The risk of infection with *L. infantum* increased significantly among dogs that were raised in shelters made of earthen floors in comparison with those that were kept in shelters with paved floors. Since paved floors are easy to clean and covered with less organic matter than soil floors, they offer unfavourable conditions for the spreading of the vector larvae [48,49].

## 5. Conclusions

The results that were obtained in this study confirm the presence of antibodies against CanL in exposed dogs in some governorates of Northern Egypt and identify the risk factors that are associated with the infection. A high *L. infantum* seroprevalence rate was observed in older, male, short-haired dogs that were kept in earthen-floor shelters, particularly in the absence of veterinary care or any application of insecticides. Thus, the results show an increased number of asymptomatic dogs that acted as reservoirs for the disease and should be considered a great risk to public health. Consequently, additional information about risk factors and application of efficient control measures is a potential tool to reduce the zoonotic hazard.

## Figures and Tables

**Table 1:** Seroprevalence of canine leishmaniasis in relation to different variables.

Factor	No of Examined Dogs	No of Positive	No of Negative	%	95% CI	Statistics
**Location**						
Cairo	95	27	68	28.4	20.33–38.19	
Giza	115	33	82	21.7	15.2–30.1	χ2 = 4.179 df = 4 *p* = 0.382
Qalyubia	75	9	66	18.7	11.5–28.9	
Kafr ElSheikh	80	12	68	18.8	11.7–28.7	
Gharbia	85	15	70	17.6	11–27.1	
**Age**						
6–12 months	30	1	29	3.3	0.2–19.1	χ2 = 11.483 df = 4 *p* = 0.02 *
1–2 years	65	9	56	13.8	6.0–25.2	
2–4 years	230	60	170	26.1	20.6–32.3	
4–6 years	95	21	74	22.1	14.5–32.0	
>6 years	30	5	25	16.7	6.3–35.4	
**Sex**						
Male	280	67	213	23.9	19.1–29.5	χ2 = 2.975 df = 1 *p* = 0.08
Female	170	29	141	17.1	11.9–23.7	
**Breed**						
German Shepherd	170	31	139	18.2	13.2–24.7	χ2 = 2.891 df = 2 *p* = 0.236
Rott Weiler	100	27	73	27	19.3–36.4	
Mongrel	180	38	142	21.1	15.7–27.6	
**Hair length**						
Long	150	21	129	14	9.1–20.8	χ2 = 7.210 df = 1 *p* = 0.007 *
Short	300	75	225	25	20.3–30.4	
**Veterinary care**						
Yes	260	32	228	12.3	8.7–17.1	χ2 = 29.891 df = 1 *p* = 0.0001 *
No	190	64	126	33.7	27.0–40.9	
**Application of insecticides**						
Yes	270	32	238	11.9	8.4–16.5	χ2 = 36.158 df = 1 *p* = 0.0001 *
No	180	64	116	35.6	28.7–43.1	
**Floor of shelter**						
Paved	310	45	265	14.5	10.8–19.1	χ2 = 27.594 df = 1 *p* = 0.0001 *
Soil	140	51	89	36.4	28.5–45.0	

* The result is significant at *p* < 0.05.

**Table 2:** Multivariable logistic analysis for risk factors of *Leishmania infantum* infection in dogs.

Variable	B ^a^	SE ^b^	OR ^c^	95% CI ^d^	*p* Value
**Age**					
1–2 years	1.602	1.094	4.96	0.6–42.3	0.143
2–4 years	2.485	1.041	12.00	1.6–92.3	0.017 *
4–6 years	2.249	1.060	9.48	1.2–75.8	0.034 *
>6 years	1.678	1.146	5.35	0.6–50.6	0.143
**Sex**					
Male	0.655	0.269	1.93	1.1–3.3	0.015 *
**Breed**					
**German Shepherd**	0.462	0.320	1.59	0.9–2.9	0.15
**Hair length**					
Short	0.729	0.285	2.07	1.2–3.6	0.011 *
**Veterinary Care**					
No	0.994	0.387	2.7	1.3–5.8	0.010 *
**Application of Insecticides**					
No	1.127	0.378	3.09	1.5–6.5	0.003 *
**Floor of shelter**					
Soil	0.353	0.373	1.42	0.7–2.9	0.343

^a^ Logistic regression coefficient; ^b^ Standard error; ^c^ Odds ratio; ^d^ Confidence interval; * The result is significant at *p* < 0.05.

## Data Availability

All data analyzed during this study are included in this published article.
